# *Opuntia* spp.: Characterization and Benefits in Chronic Diseases

**DOI:** 10.1155/2017/8634249

**Published:** 2017-04-09

**Authors:** María del Socorro Santos Díaz, Ana-Paulina Barba de la Rosa, Cécile Héliès-Toussaint, Françoise Guéraud, Anne Nègre-Salvayre

**Affiliations:** ^1^Facultad de Ciencias Químicas, Universidad Autonoma de San Luis Potosí, San Luis Potosí, SLP, Mexico; ^2^Instituto Potosino de Investigación Científica y Tecnológica (IPICyT), San Luis Potosi, SLP, Mexico; ^3^Toxalim (Research Center in Food Toxicology), INRA, ENVT, INP-Purpan, UPS, Toulouse, France; ^4^University of Toulouse, Toulouse, France; ^5^INSERM U 1048, Toulouse, France

## Abstract

*Opuntia* species have been used for centuries as food resources and in traditional folk medicine for their nutritional properties and their benefit in chronic diseases, particularly diabetes, obesity, cardiovascular diseases, and cancer. These plants are largely distributed in America, Africa, and the Mediterranean basin. *Opuntia* spp. have great economic potential because they grow in arid and desert areas, and *O. ficus-indica*, the domesticated *O*. species, is used as a nutritional and pharmaceutical agent in various dietary and value-added products. Though differences in the phytochemical composition exist between wild and domesticated (*O. ficus-indica*) *Opuntia* spp., all *Opuntia* vegetatives (pear, roots, cladodes, seeds, and juice) exhibit beneficial properties mainly resulting from their high content in antioxidants (flavonoids, ascorbate), pigments (carotenoids, betalains), and phenolic acids. Other phytochemical components (biopeptides, soluble fibers) have been characterized and contribute to the medicinal properties of *Opuntia* spp. The biological properties of *Opuntia* spp. have been investigated on cellular and animal models and in clinical trials in humans, allowing characterization and clarification of the protective effect of *Opuntia*-enriched diets in chronic diseases. This review is an update on the phytochemical composition and biological properties of *Opuntia* spp. and their potential interest in medicine.

## 1. Introduction

Long historical and worldwide use of medicinal plants and phytochemicals has demonstrated the efficacy of traditional medicine to prevent the onset and progression of chronic diseases. In Mexico, among the number of plants identified and used in folk medicine, *Opuntia* species (spp.) exhibit a lot of beneficial properties and high biotechnological potential. They grow in dry desert area where hard environmental conditions prevail, and they have been used for centuries as food resources and in folk medicine for the treatment of chronic diseases (obesity, cardiovascular and inflammatory diseases, diabetes, and gastric ulcer) and many other illnesses [1]. Some species such as *O. ficus-indica* are cultivated for economical and medicinal purposes in Mexico area. Though differences in the phytochemical composition exist between domesticated and wild *Opuntia* spp., the presence of antioxidants (flavonoids, ascorbate), pigments (carotenoids, betalains such as indicaxanthin), or phenolic acids has been reported in all *Opuntia* products, including seeds, roots, pears, cladodes, or juice. Antioxidants could be responsible for the nutritional and protective benefit of *Opuntia*-enriched diets in chronic diseases, in which inflammation and oxidative stress play a major involvement. Other plant materials such as biopeptides or soluble fibers have been characterized and contribute to the medicinal properties of *Opuntia* spp. This review is an update on the active compounds and the biological and medical benefit of wild and domesticated *Opuntia* spp. in chronic diseases.

## 2. *Opuntia* History and Production

### 2.1. *Opuntia* History

Plants are classified as succulent when they exhibit pronounced water storage in one or more organs. The Opuntioid cacti represent the most spectacular species of succulent plants, which are characterized by a shallow root system that permits rapid water uptake; a thick, waxy cuticle that prevents excessive water loss; and crassulacean acid metabolism (CAM), an alternative photosynthetic pathway, that allows plants to uptake atmospheric CO_2_ at night when water loss is minimized [2]. Opuntioid cacti are recognized as ideal crops for arid regimes because they are extremely efficient at generating biomass under water-deficient conditions [3].

*Opuntia* spp. is one of the most diverse and widely distributed genus in America [4], but the highest richness of wild species are found in Mexico, as at least 126 species with different degrees of domestication have been observed [5]. There are evidences that during the process of *Opuntia* domestication, the continuous and systematic gather of cladodes and fruit favored the development of exceptional features, with the purpose to adapt plants to successfully live in human-made environment and maximize yield or any given selected feature [6, 7].

Wild species of the *Opuntia* genus have been grouped in one line of domestication processes where the wildest species are *O. streptacantha* and *O. hyptiacantha*. Others species are semidomesticated like *O. megacantha* and *O. albicarpa* [5]. As a result of domestication, the *Opuntia* fruit has been enhanced in flavor, size, shape, pulp texture, and decreased seed hardness and quantity. Regarding cladodes, changes occurred in shape, color, earliness, flavor, texture, and mucilage quantity and quality [5]. *O. ficus-indica* is a long-domesticated cactus crop that is important in agricultural economies throughout arid and semiarid parts of the world. It has been proposed that *O. ficus-indica* is a spineless cultivar derived from *O. megacantha*, a native species from central Mexico [8, 9]. Bayesian phylogenetic analysis of nrITS DNA sequences indicates that the center of domestication for this species is located in central Mexico [10].

The easy of clonal *Opuntia* propagation probably explains why it is easily distributed worldwide. Evidence exists for the use of *Opuntia* as human food since at least 9000 years ago [11] or even as early as 12,000 years ago [8, 12].

In the recorded history of the Old World, *O. ficus-indica* was certainly known at the beginning of the 16th century [6, 7] and it is believed that this species accompanied Columbus in his first return to Lisbon in 1493 [4], placing *O. ficus-indica* in the Caribbean by at least the late 1400s. The plants are also recorded in cultivation in Tlaxcala, Mexico, in 1519. *O. ficus-indica* fruits and shoots were also reportedly consumed by the Maya of southeastern Mexico [8]. There is also some evidence for the use of *O. ficus-indica* by the Nazca of Peru [13]. The succulent, ever-fresh cladodes were certainly a novelty to late 15th century and later Europeans [14].

Actually, *O. ficus-indica* is as important as corn and tequila agave in the agricultural economy of modern Mexico [15] and represents important food and feed resources. Its economic importance has gradually increased around the world as a health-promoting food [16]. *O. ficus-indica* is grown for the large, sweet fruits (often called “tunas”), which are available in local and commercial markets worldwide [17]. In addition, the young cladodes (stem segments) of *O. ficus-indica* are harvested as a vegetable crop (often called nopalitos). Other uses have been reported, including as a binding and waterproofing agent in adobe and its medicinal properties [18, 19]. *O. ficus-indica*, along with other Opuntia and Nopalea species, has been grown from pre-Columbian times as a host plant for cochineal insects (*Dactylopiuscoccus*) for the production of valuable, vivid red and purple dyes [4, 20].

Interest in health care among consumers is increasing steadily and has expanded to dietary intake, and as a result, the food industry has started to produce new food types based on “nopalitos” to reflect this change in consumerism [18].

### 2.2. *Opuntia* Production

*Opuntia* plants produce edible stems known as pads, vegetable, cladodes, nopales, or pencas. The tender young part of the cactus stem, young cladode or “nopalitos”, is frequently consumed as vegetable in salads, while the cactus pear fruit is consumed as a fresh fruit. Mexico and Italy are the main producer countries and consumers, from the approximately 590,000 ha cultivated worldwide, México accounts for 70% and Italy for 3.3%. Under optimal conditions, the annual production in Mexico can reach 350,000 tons [5].

Nowadays, *Opuntia* plants are grown in more than 30 countries; Chile and South Africa are producers with 1500 ha and 1000 ha, respectively. Israel and Colombia account for with 300 ha cultivated. In the US, California is the leading state with 200 ha cultivated producing 4000 tons of dry matter.

## 3. Cladodes Chemical Composition

*Opuntia* spp. have a high nutritional value, mainly due to their mineral, protein, dietary fiber, and phytochemical contents [21].  Table 1 indicates the chemical composition of some wild and domesticated species (*O. ficus-indica*). The main constituent of *O. ficus-indica* cladodes is water (80–95%), followed by carbohydrates (3–7%), fibers (1-2%), and proteins (0.5–1%). However, the chemical composition of cladodes is modified by maturity stage, harvest season, environmental conditions, postharvest treatment, and type of species [22–24]. In some wild species such as *O. robusta* (Tapon) and Blanco, 17.4 to 19% proteins can be reached [22]. *O. leucotricha* (Duraznillo) yield high-quality cladodes, since the pericarp can be easily removed and will neither fall apart during boiling nor release mucilage [21].

It is well known that *Opuntia* cladodes are a good source of dietary fibers [25], which may help in reducing body weight by binding to dietary fat and increasing its excretion [26]. This may explain why cladodes are considered as hypolipidemic.

*Opuntia* cladodes contain higher calcium (Ca) contents relative to vegetables, fruits, and nuts [24, 27, 28].  Table 2 shows the mineral composition of wild and domesticated *Opuntia* spp.

Calcium content seems higher in *Opuntia* spp. than in other plants such as spinach (1151 mg/100 g), lettuce (703 mg/100 g), cabbage (511 mg/100 g), and broccoli (43 mg/100 g). Aguilera-Barreiro et al. [29] reported that consumption of cactus improves the bone mineral density in women with low bone mass.

The *Opuntia* spp. cladodes are also widely known for their strong viscous materials and hydrophilic polysaccharides of large molecular weight due to their great capacity to absorb and retain water [21]. The soluble dietary fiber and calcium contents of *Opuntia* spp. cladodes are considered to be very important elements for their physical properties because the main cell wall polysaccharide consists of low methoxyl pectin [19].

### 3.1. Total Phenolic and Flavonoid Content in *Opuntia* spp

It is well known that the secondary metabolite accumulation depends on biotic and abiotic factors. Since *Opuntia* spp. used in this study were cultivated under the same environmental conditions, then the differences in the amount of metabolites should be related to each species biochemical characteristics.  Table 3 shows the content of phenolic acids and flavonoids present in wild and domesticated *Opuntia* spp.

The beneficial properties of *Opuntia* spp. are related to their content in chemical compounds as minerals, polyphenols, vitamins, polyunsaturated fatty acids, and amino acids, as recently reviewed by El-Mostafa et al. [30]. *O. ficus-indica* is the most domesticated and studied species, and several reports describe the main compounds found in cladodes, flowers, and fruits [21, 30, 31]. However, other *Opuntia* spp. used in folk medicine are important sources of bioactive compounds. In this chapter, we report the antioxidant composition (phenolic acids, flavonoids, betalains, and vitamins) of wild *Opuntia* spp. and the most recent information related to *O. ficus-indica*.

#### 3.1.1. Phenolic Compounds

The phenolic compounds are important antioxidants since phenoxy radical intermediates (PO) are relatively stable due to resonance and act as terminators of propagation route by reacting with other free radicals. On the other hand, the phenolic hydroxyl groups can donate a hydrogen atom or an electron to a free radical conferring radical scavenging activities. They also extended conjugated aromatic system to delocalize an unpaired electron. Some phenolic compounds with dihydroxy groups can conjugate transition metals, preventing metal-induced free radical formation [32].

The total phenolic compound content in *Opuntia* spp. is quite variable and is affected by the maturity stage, harvest season, environmental conditions, postharvest treatment, and species (Table 3). It has been reported that *O. ficus-indica* fruits contain 218 mg GAE/100 g FW [30], but the wild species *O. stricta* also exhibited high concentrations of these metabolites (204 mg GAE/100 g FW), followed by *O. undulata*, *O. megacantha*, *O. streptacantha*, and *O. dinellii* (164.6, 130, 120, and 117 mg/100 g FW pear, respectively) [33–36]. Important differences in the content of flavonoids among species are described, ranging between 9.8 (in *O. stricta*) and 50.24 (in *O. megacantha*) mg QE/100 g FW [33, 34].

In relation to the color of fruits, variations on metabolite contents can be observed. Purple fruits of *O. ficus-indica* cultivated in Italy, Spain, USA, Tunisia, and Saudi Arabia contain higher levels of phenolics (89–218.8 mg GAE/100 g FW) than the orange fruits (69.8 mg GAE/100 g FW) [33, 37–40]. However, the Mexican cultivars, *O. megacantha* (orange fruits), *O. streptacantha*, and *O. robusta* (purple fruits), exhibited a similar phenolic compound concentration (120–140 mg GAE/100 g FW) [34].

Pads are also source of polyphenols. It has been reported that *O. violacea*, *O. megacantha*, *O. atropes*, and *O. albicarpa* contain high concentration of phenolic acids (17.8–20 mg GAE/g DW), while *O. rastrera* and *O. undulata* present the lowest values (0.39–0.95 mg GAE/g DW) [22]. A recent comparative analysis realized with 15 *Opuntia* cultivars from *O. streptacantha*, *O. hyptiacantha*, *O. megacantha*, *O. albicarpa*, and *O. ficus-indica*, cultivated under the same environmental conditions and at the same developmental stage, showed that metabolite content in cladodes was independent of domestication grade. Thus, differences depend basically on the biochemical characteristics of each species [28].

Flowers and peels could exhibit a higher phenolic content than fruits and pads with about 45.7 g/100 g FW, so it is recommended to exploit these materials to obtain biocompounds with antioxidant characteristics [30, 36].

The phenolic profile in *Opuntia* is complex with more than 30 compounds identified in cladodes of different species [21, 22, 28, 31], more than 20 in seeds, and 44 compounds in juices [41, 42]. The most common compounds present in *Opuntia* tissues from wild and cultivated species include kaempferol, quercetin, isorhamnetin, and isorhamnetin glucosides (Table 3). Kaempferol 3-O-arabinofuranoside was detected only in *O. streptacantha*, quercetin 3-O-rhamnosyl-(1-2)-[rhamnosyl-(1-6)]-glucoside was detected only in *O. ficus-indica* cladodes [28], and isorhamnetin-3-O-rutinoside was present in the juice and peel from *O. dilleni* [36]. The rare piscidic acid and derivatives, restricted to plants exhibiting crassulacean acid metabolism and succulence, were detected in juices from *O. ficus-indica* [42]. In seeds, sinapoyl-glucose, sinapoyl-diglucoside, three isomers of feruloyl-sucrose, catechin, rutin, and quercetin derivatives were detected [43]. Taurine, an unusual sulfonic acid, was identified in Sicilian and African cultivars [31].

#### 3.1.2. Betalains

Betalains are water-soluble molecules with two or three nitrogen atoms and about 55 structures known, including the red-violet betacyanins and the yellow-orange betaxanthins. Their characteristic is the N-heterocyclic nature with betalamic acid being their common biosynthetic precursor. Aldimine formation with *cyclo*-Dopa yields the betanidin aglycone that is usually conjugated with glucose and sometimes with glucuronic acid. On the other hand, betaxanthins are conjugates of betalamic acid with amino acids or amines [44].

Betalains are excellent radical scavengers with an antioxidant activity 3-4 times higher than ascorbic acid, rutin, and catechin [45], twice higher than that measured for pear, apple, tomato, banana, and white grape, and from the same order as pink grapefruit, red grape, and orange [46]. The monophenol nature of betanin and reducing intermediates during the oxidation process may confer to the molecule a higher H-atom or electron donation potential. In the case of betaxanthins, the antioxidant power has been linked to the presence of one or two phenolic hydroxy groups in their structure. Betacyanins also have a potential to inhibit NO or nitrogen radical species due to the presence of a catechol group in betanidin structure [47, 48].

In the literature, it is reported that fruits of cactus pear contain different betalains whose concentration depends on species, cultivar, and geographic region. The betacyanins identified in *Opuntia* fruits include betanidin, betanin, isobetanin, isobetanidin, neobetanin, phyllocactin, and gomphrenin I [3, 4, 24–26]. *O. streptacantha* (Cardona cultivar), (Rojalisa cultivar), and *O. megacantha* (Naranjona cultivar) contain traces of betanidin 5-O-*β*-sophoroside [34].

The presence of conjugates of betalamic acid with several amino acids is reported in pears, corresponding to miraxanthine II (aspartic acid), indicaxanthin (proline), vulgaxantin I (glutamine), vulgaxantin II (glutamic acid), vulgaxantin III (asparagin), vulgaxantin IV (leucine), portulacaxanthin I (tyrosine), portulacaxanthin III (lysine), ɣ-aminobutyric acid-betaxanthin, serine-betaxanthin, valine-betaxanthin, isoleucine-betaxanthin, isoproline-betaxanthin, phenylalanine-betaxanthin, histidine-betaxanthin, phenethylamine-betaxanthin, and muscaaurin [42, 46–52]. Using cactus pears as a betalain source is of great interest because they are highly flavored, with better nutritional properties than red beetroot.

### 3.2. Vitamins

The main vitamins present in *Opuntia* spp. include vitamin E, vitamin C, vitamin K, and tocopherols [30]. The vitamin levels depend on the cultivar type. In *O. ficus-indica*, Italian varieties exhibit values ranging 30–36 mg Vit C/100 g FW in fruits [40, 49, 52]; in *O. ficus-indica* and *O. streptacantha* (red-skinned), *O. stricta* (yellow-skinned), *O. undulata, O. dinellii*, and *O. lindheimeri* (purple-skinned) cultivated in Texas, the values range from 12.1 to 81.5 mg Vit C/100 g, while in *O. dillenii* and *O. ficus-indica* cultivars from Tenerife, the concentration ranged from 17 to 29.7 mg/100 g, respectively [35, 37]. Usually, the highest ascorbic acid content is present in red-skinned fruits (815 mg/g FW) and the highest carotenoid content in yellow-skinned fruits (23.7 mg/g FW) [35].

Although other compounds present in the various *Opuntia* parts may exhibit antioxidant properties, most reports suggest that polyphenols, betalains, and vitamins are the main compounds involved in their biological properties. Beside the use of pads or fruits, the seeds and peels have a high promising potential for the development of new nutraceutic products.

## 4. Biological and Medical Properties of *Opuntia* spp. in Chronic Diseases

*Opuntia* extracts have been used since centuries for nutritional and medical purposes, and their therapeutical interest has recently been made clear by in vitro and in vivo scientific studies [30]. We report here the protective properties of various *Opuntia* spp. in the development of atherosclerosis and cardiovascular diseases, diabetes, obesity, and cancer.

### 4.1. *Opuntia* spp. in Cardiovascular Diseases

Atherosclerosis and its related cardiovascular complications are the leading worldwide cause of death related to chronic diseases [53]. Since the beginning of the last decade, even if the cardiovascular mortality rates tend to slightly decline in western countries, they are fastly increasing in the developing world. In Mexico, the mortality due to coronary artery diseases (CAD) strongly increased in the last 30 years, representing more than 11% of deaths in the country by 2006 [54]. The lifelong risk factors for CAD in Mexico have been studied and are similar to those reported in Western countries, that is, hypertension, high cholesterol levels, smoking, diabetes, and obesity [55, 56]. Interestingly, CAD and risk factors such as diabetes were rare in Mexico before the second half of the twentieth century, suggesting that lifestyle changes including nutritional habits have contributed to the increased cardiovascular risk in this country [56]. Strategies focusing on changing lifestyles are thus becoming a priority, with particular focus on smoking and dietary habits. In this context, there is an increasing interest for the nutritional benefit of *Opuntia* spp. to prevent the development of CAD. The antiatherogenic properties of *Opuntia* spp. result from their high antioxidant (polyphenols) content which could decrease lipid peroxidation, an important risk factor in atherosclerosis [57], and also from dietary fibers and proteins, which exhibit lipid-lowering properties, in humans and in animals.

#### 4.1.1. Cholesterol-Lowering properties of *Opuntia*

Several reports point out the antioxidant and antiatherogenic properties of *Opuntia* spp. [57]. First, the consumption of *Opuntia* juice and fruits naturally prevents oxidative stress and improves the redox status in healthy humans [58]. Budinski et al. [59] reported that the regular consumption of prickly pears from *O. robusta,* by patients affected with familial heterozygous hypercholesterolemia, significantly lowered the plasma levels of LDL cholesterol and the plasma and urine content of 8-epi-prostaglandin F_2_*α*, a F2 isoprostane produced through the peroxidation of arachidonic acid. No modifications were observed on HDL and triglycerides [59]. Likewise, the consumption by women affected with metabolic syndrome, of dried leaves from *O. ficus-indica* as dietary supplement, showed a rapid increase in circulating HDL cholesterol level concomitantly with a decrease in LDL cholesterol and (slightly) in triglycerides, indicating that the plant exerts an hypocholesterolemic effect [60]. These lipid-lowering properties were confirmed by studies on mice fed with a hypercholesterolemic diet. When the animals were supplemented with a methanolic extract from *O. joconostle* (polyphenol enriched) seeds, they exhibited a marked reduction in circulating LDL cholesterol and triglyceride levels, by comparison with animals fed with a placebo [61].

The lipid-lowering properties of *Opuntia* spp. are not well clarified. Antioxidants block lipid peroxidation, but have usually no effect on plasma lipid profiles, except grape polyphenols (such as resveratrol), which decrease plasma triglyceride levels and alter the metabolism of VLDL [62]. In *Opuntia*, the lipid-lowering properties may rather result from their content in dietary fibers, as supported by data from Wolfram et al. [63]. These authors reported that prickly pears from *O. robusta* lower the cholesterol levels in hyperlipemic nondiabetic human patients. They concluded that the protective effect of *Opuntia* prickly pear may result from pectin, a soluble fiber [63]. The mechanism elicited by pectin could evoke an alteration of hepatic cholesterol metabolism without affecting cholesterol absorption [64, 65]. Likewise, glycoprotein isolated from *O. ficus-indica* var. *saboten* MAKINO (an *Opuntia* variety used in folk medicine in Korea) exerts potent antioxidant and hypolipidemic properties evidenced by a protective effect on mice treated with triton WR-1339, an inhibitor of lipoprotein lipase [66]. It is to note that the ingestion of *Opuntia* prickly pears also improves the platelet function and haemostatic balance, thus contributing to prevent the atherosclerotic risk [63].

#### 4.1.2. Antiatherogenic Properties of *Opuntia* spp

The early stages of atherosclerosis development are characterized by the retention in the intima of LDL, which undergo oxidation upon the attack of their polyunsaturated fatty acid content by environmentally produced reactive oxygen species (ROS), [67–70]. Several ROS-producing systems are involved in the LDL oxidation process, among them the activation of NADPH oxidase NOX2 [71], through a mechanism implicating the scavenger receptor LOX-1 [72]. Oxidized LDL initiate inflammatory processes in the vascular wall, leading to the recruitment of monocytes/macrophages and the accumulation of foam cells, and finally to the formation of the fatty streaks which are the early atherosclerotic lesions [69, 70]. Beside their role in fatty streak formation, oxidized LDL behave as strong cytotoxines for vascular cells [70, 73]. Lipid oxidation products (LPO) formed during the onset of LDL oxidation are detected responsible for the proapoptotic and proinflammatory properties of oxidized LDL [70, 74]. Furthermore, aldehydic LPO such as hydroxynonenal (HNE), malondialdehyde (MDA), or acrolein form adducts on proteins generating their progressive dysfunction and contributing to inflammation and apoptosis [74].

Most antioxidants are antiatherogenic as they neutralize the formation of ROS by vascular cells and exhibit anti-inflammatory and antiapoptotic properties against the effects of oxidized LDL on vascular cells [70]. Our group recently compared the antiatherogenic properties of *Opuntia* powders obtained from the cladodes of five different wild spp. (*O. streptacantha* var. *cardona*, *tuna loca*, *O. hyptiacantha*, and *O. megacantha*), medium (*O. albicarpa*), and domesticated (*O. ficus-indica*) [75]. Precisely, the effect of cladodes was tested on oxidation of LDL evoked by murine endothelial cells (an in vitro model mimicking the mechanism of LDL oxidation occurring in vivo in the vascular wall). Cladode powdered and solubilized in the culture medium dose-dependently inhibited LDL oxidation and the subsequent formation of foam cells by macrophages, which suggests that *Opuntia* spp. could inhibit the early steps of atherogenesis [75]. This inhibitory effect of *Opuntia* spp. involves an inhibition of NADPH oxidase (NOX2) resulting in a decreased generation of intracellular and extracellular superoxide anion (O_2_°-), a main ROS involved in the LDL oxidation process [75, 76]. No major difference of protection was observed between wild and domesticated *Opuntia* spp. Likewise, *Opuntia* spp. inhibit the nuclear translocation of the redox-sensitive transcription factor NF*κ*B and the subsequent expression of ICAM-1 and VCAM-1 adhesion molecules [76, 77] and thus exhibit anti-inflammatory properties resulting from their inhibitory effect on cellular ROS production. Additionally, wild *O. streptacantha* and domesticated *O. ficus-indica* inhibit the toxicity of cell-oxidized LDL [75, 76] and oxidized lipids such as 7-ketocholesterol [78] through mechanisms implicating an inhibition of intracellular oxidative stress and subsequent cytosolic calcium deregulation [78].

In vivo studies on apoE-KO mice, which spontaneously develop atherosclerotic lesions in basal diet conditions, indicated that the supplementation of the diet in *O. streptacantha* or *O. ficus-indica* powdered cladodes (10 mg/kg during 15 weeks) significantly reduced the development of atherosclerotic lesions [76]. In addition, the lowering effect of *Opuntia* spp. on LDL oxidation was supported by a decrease in HNE-adduct accumulation in the intima [76]. In contrast to the data reported by Osorio-Esquivel et al. [61], the intake of *O. streptacantha* or *O. ficus-indica* cladodes did not reduce the plasma cholesterol level [56]. This discrepancy may result from the diet (basal or cholesterol-enriched) or from the *Opuntia* components (cladodes versus seeds). Nevertheless, both lipid-lowering and antioxidant properties of the different wild and domesticated *Opuntia* spp. may support their efficacy to prevent or slow down atherosclerotic lesion development and subsequent cardiovascular diseases.

### 4.2. *Opuntia* spp. in Diabetes

Type 2 diabetes mellitus (T2DM) is a multifactorial disease including genetic determinants of individual susceptibility and environmental lifestyle factors. It is considered as a major health problem worldwide, with an increasing incidence and invalidating long-term complications, affecting macro- and microvasculature, kidney, heart, nerves, or eyes [79].

Several reports in diabetic patients and animals point out the antihyperglycemic and antihyperinsulinemic properties of *Opuntia* spp. The dietary intake of nopal (*O. ficus-indica*) improves the postprandial response of glucose, insulin, glucose-dependent insulinotropic peptide (GIP) index, and the glucagon-like peptide 1 (GLP-1) index on T2DM patients after consumption of a high-carbohydrate or high-soy-protein breakfast [80]. The hypoglycemic efficacy could be higher after heating *Opuntia* extracts, as reported by Frati-Munari et al. [81], in patients consuming broiled *O. streptacantha* extracts. Likewise, glycemia and glycated hemoglobin are lowered to normal values in rats rendered diabetic after treatment by streptozotocin (STZ) and supplemented with an extract of *O. fuliginosa* prickly pear [82]. Likewise, *O. humifusa* stems promote a blood glucose and cholesterol decrease in STZ-treated rats [83].

In a recent study, Andrade-Cetto et al. [84] indicated that *O. streptacantha* extracts do not reduce glycemia in STZ-treated rats when compared to the control but exhibit an antihyperglycemic effect when administered before an oral glucose tolerance test (OGTT). A similar result was observed in obese prediabetic patients (men and women) treated for 16 weeks with OpunDia™ (a mixture of *O. ficus-indica* cladode and fruit extracts), that is, a net blood glucose decrease when the mixture was given before OGTT, suggesting that *Opuntia* spp. intake may reduce blood glucose in postprandial conditions [85].

The hypoglycemic mechanism evoked by *Opuntia* spp. ingestion could be due to dietary fibers such as pectin and mucilage [81], which may slow down the absorption of glucose by increasing the viscosity of food in the gut [86, 87]. Likewise, polysaccharides isolated from *O. ficus-indica* or *O. streptacantha* could exert a hypoglycemic effect in diabetic mice [88]. The hydrolysis of disaccharide has been proposed to explain the hypoglycemic effect of *O. streptacantha*, via an inhibition of *α*-glucosidase inhibitors (AGIs) or through a barrier function. However, this hypothesis was not verified [89]. Another hypothesis is that *Opuntia* spp. stimulate insulin secretion via a direct action on pancreatic beta cells [90], a mechanism also observed after exercise in healthy subjects and involved in fast glycogen resynthesis [91]. In addition, the antioxidant properties of *Opuntia* spp. could play a role in the prevention of cardiovascular complications due to T2DM. Indeed, oxidative stress plays a pivotal role in the pathophysiology of T2DM, particularly in the development of accelerated atherosclerosis lesions and cardiovascular diseases which represent a main complication in diabetes [92]. *Opuntia* spp. may exert an inhibitory effect on the oxidative environment generated by hyperglycemia, via their antioxidant components, and as suggested by a recent report by Berraaouan et al. [93] who showed that cactus pear seed oil from *O. ficus-indica* L. Mill. prevents the development of alloxan-induced diabetes in mice by quenching the generation of ROS. Though the protective mechanisms are not yet clarified, all these reports confirm that *Opuntia* spp. extracts exhibit antihyperglycemic and antidiabetic properties.

### 4.3. *Opuntia* spp. in Obesity

Obesity is becoming a major public health concern all over the world. Worldwide obesity has more than doubled since 1980 with approximately 66 million obese individuals in the world [94]. The obesity prevalence has increased significantly as a result of rapid urbanization and improvement in socioeconomic conditions. In Mexico, the prevalence of obesity in adults is 32% (with a higher prevalence in females) and is about 15% in children. Mexico faces a challenging situation with one of the highest and most rapidly increasing prevalence of obesity. Thus, researchers, clinicians, and people looking to reduce body weight are always in search of solutions.

The complex pathogenesis of obesity indicates the need of different intervention strategies to confront this problem with a simple drug therapy that is more acceptable to patients. It is difficult for patients to follow diets and exercises that would improve their symptoms. Therefore, investigation of new efficient agents is an important medical field for research. Herbal supplements and diet-based therapies for weight loss are among the most common, complementary, and alternative medicine modalities. The demand for weight-loss products based on plants has increased during the last decade. This demand clearly indicates that medicinal plants for the treatment of obesity represent a current topic of interest. *O. ficus-indica* fruits, stems, seeds, and cladodes have been traditionally used in folk medicine to prevent and cure chronic diseases. Therefore, clinical pharmacologic interest in the efficacy and safety of the phytochemicals present in the genus *Opuntia* has grown during recent years due to the realization that many people self-medicate using this plant [30, 95, 96]. Different approaches can be used, including in vitro on cellular models, in vivo by the use of animal models such as mice or rats fed diets enriched with *Opuntia* extracts, and human clinical trials.

#### 4.3.1. Cellular Models for In Vitro Analysis of *Opuntia* Effects

Adipogenesis is a complex process that includes coordinated changes in adipocytes morphology, hormone sensitivity, and gene expression. Adipocytes play a central role in the maintenance of lipid homeostasis and energy balance by storing triglycerides or releasing free fatty acids in response to changes in energy demand. Obesity is not only caused by adipose tissue hypertrophy but also by adipose tissue hyperplasia, which triggers the transformation of preadipocytes into adipocytes. Adipocyte dysfunction is strongly associated with the development of obesity. Many studies have shown that *Opuntia* extracts such as the flavonoid kaempferol or isorhamnetin can suppress lipid accumulation or inhibit adipogenesis through adipogenic-responsible genes downregulation [97, 98]. The use of cellular models allows a better knowledge of the gene regulations and the metabolic pathways in which plant extracts could interfere.

#### 4.3.2. Animal Models

The use of animal models with diet-induced obesity helped to evaluate the nutritional values and some biological parameters of cactus seed supplements [99, 100], dehydrated *O. ficus-indica* cladodes [101, 102], or a combination of pre-Hispanic Mexican diet including nopal and nopal seeds [103] on rat models. These studies have shown that supplementation of diet with *O. ficus-indica* seed powder may have a favorable effect on the serum lipid profile and glucose, linked with beneficial effects on atherosclerosis, diabetes, and obesity [99, 100]. It exerts a favorable impact on insulin sensitivity through the regulation of genes involved in adipocyte differentiation [102], it attenuates hepatic steatosis in obese Zucker (fa/fa) rats [101], and it decreases metabolic and cognitive abnormalities and gut microbiota dysbiosis caused by a high-fat diet in rats [103]. To determine the metabolic effect of an *O. ficus-indica* extract on a mouse model of diet-induced obesity [104], the extract was added to a high-fat diet and administered to mice for 12 weeks. Mice fed with the high-fat diet supplemented with *O. ficus-indica* extract gained less body weight and exhibited significantly lower circulating cholesterol, LDL cholesterol, and HDL cholesterol, when compared to mice fed with the high-fat diet alone. In this study, the *O. ficus-indica* extract prevented the development of metabolic abnormalities associated with diet-induced obesity [104]. Thus, the use of different animal models provided many clues to the potential effects of *Opuntia* extracts in terms of energy metabolism, gene regulation, and insulin and glucose pathways regulation, suggesting that cactus pears, given in different ways in the diet, could be efficient in human treatment of obesity.

#### 4.3.3. Clinical Trials

Antiobesity agents obtained from natural products are gaining more and more interest in the scientific community, and some of their active compounds have reached clinical trials.

In their double-blind, randomized, placebo-controlled clinical investigation, Grube et al. [105] used Litramine, a natural fiber complex derived from *O. ficus-indica*, associated with a hypocaloric diet, plus moderate physical activity (30 minutes walking or cycling). In a 12-week treatment on a panel of 125 overweight and obese volunteers, they were able to show a weight loss of at least 5% of the volunteers' initial body weight compared to placebo. They showed significantly greater reduction in BMI (body mass index), body fat composition, and waist circumference. Importantly, they noticed that Litramine fibers complement was well tolerated and that no adverse reactions were reported. These results suggest that the natural fiber complex Litramine can be effective in promoting weight loss. To go further, Uebelhack et al. [26] and Chong et al. [106] determined that *Opuntia*-derived fibers bind to dietary fat and increase its excretion and thus reduce its absorption; this leads to a lower energy intake and weight loss. The safety assessment also revealed minimal concerns as Litramine is well tolerated, unlike lipase inhibitors usually used as weight loss complements acting on the inhibition of enzymes responsible for the digestion of long-chain triglycerides that present gastrointestinal negative side effects up to possible liver damages. Another point to notice is that acute and chronic effects of OpunDia induced a significant decrease in blood glucose concentrations after acute administration of 400 mg OpunDia 30 minutes before a 75 g glucose load. In the chronic phase of the study, supplementation of OpunDia for 16 weeks significantly lowered glucose concentrations (as described above), supporting the use of *O. ficus-indica* for blood glucose management [85].

The meta-analysis from Onakpoya et al. [107] reveals that even if many works report positive effects concerning *Opuntia*, whatever the mode of administration, randomized clinical trials do not indicate that supplementation with *O. ficus-indica* generates statistically significant effects on body weight and waist circumference. These conclusions may be due to the inconsistent quality of recording and high heterogeneity observed in some analyses, which makes the meta-analysis difficult to interpret. However, the results also suggest that *O. ficus-indica* ingestion results in significant reduction in body mass index, body fat percentage, and circulating triglycerides. Thus, larger well-controlled randomized clinical trials examining the effects of *O. ficus-indica* on body composition and metabolic parameters are required to conclude on the effects on body parameters. However, consumption of fruits is widely recommended for healthy lifestyle, and intake of cactus pears takes part in a well-balanced diet. This type of dietary recommendations could be adapted to different ethnic groups by incorporation of native food, known for a long time to have beneficial medical properties. Indeed, individuals at risk for diabetes, obesity, or cardiovascular diseases will prefer to include local, beneficial food in their diet as a way to improve the biochemical and clinical abnormalities associated with metabolic syndrome [108]. To achieve the potential benefits associated with the concept of local beneficial food, it should include an approach taking into account the genetic variation of the population as nutrigenetics [108].

Finally, in many countries, these “antiobesity agents” are marked as food supplements, which are exempted of strict licensing regulations routinely imposed on synthetic drugs or medicinal products before releasing them onto the market. Then, the abuse and overdose of these products are common practices, as consumers believe that increasing consumption of these products will increase weight loss and treatment efficacy [96].

Thus, even if the meta-analysis of Onakpoya et al. [107] reveals a need for more studies to be conclusive, it seems that the genus *Opuntia* is rich in healing properties and could be of beneficial interest in weight loss and possibly in dealing with chronic diseases such as metabolic syndrome.

### 4.4. *Opuntia* spp. in Cancer

Numerous studies have demonstrated the cytotoxic effects of various parts of *Opuntia*, namely the prickly pears (fruits), with or without peels and seeds, the cladodes or stems, and even the roots, on cancerous cell lines.

Antunes-Ricardo et al. [109] evaluated the cytotoxic effects of *O. ficus-indica* cladode flour extracts (var. Jalpa) or of purified isorhamnetin glycosides on two models of human colon cancer cell lines, namely, HT-29 and Caco2, representing apoptosis-resistant and apoptosis-susceptible cell lines, respectively, while normal fibroblasts (NIH 3T3) were used as controls. These authors reported that cladode flour extract and purified isorhamnetin glycosides were more cytotoxic to HT-29 cells than to Caco2 or to controls, with an effect of the glycosylation pattern. These effects were related to apoptosis induction through caspase cascade, which plays a central role in apoptosis pathways. Naselli et al. [110] studied the effect of *O. ficus-indica* fruit aqueous extract and its betalain pigment indicaxanthin on the proliferation of the human colon cancer cell line Caco2. These authors showed a dose-dependent apoptotic effect on proliferating cells, while no effect was reported on differentiated cells. In this study, indicaxanthin presented an epigenomic effect on the tumor suppressor gene p16INK4a, through de-methylation of its promoter and activation of its expression. Sreekanth et al. [111] reported that betanin, extracted from *O. ficus-indica* fruits, was able to inhibit the growth of the human chronic myeloid leukemia cell line K562, through apoptotic intrinsic pathway.

Chavez-Santoscoy et al. [112] tested the cytotoxic effect of filtered juices from prickly pears of various species of *Opuntia* on several cancer lines. The PC3 prostate and the Caco2 colon cell lines were the most affected, while the growth of the mammary MCF-7 and the hepatic HepG2 cell lines was diminished at a lesser extent. Normal fibroblasts were used as controls. The most cytotoxic species on cancer cells was *O. rastrera rastrero* that presented at the same time the best antioxidant content and capacity among the various species tested. In contrast, Kim et al. [113] showed that extracts from *O. humifusa* cladodes were able to induce apoptosis in MCF-7 cells and human colon SW-480 cells. Water-partitioned fractions of fruits and stems of *O. humifusa* were reported to suppress the growth of U87MG glioblastoma cells, in association to an increase in ROS production in the cells [114]. The same team reported a similar effect on HeLa cervical carcinoma cells, while normal fibroblasts were unaffected [115]. Serra et al. [116] showed that polyphenol-rich juice concentrates of various *Opuntia* were cytotoxic to HT-29 colon cancer cell lines, but not to Caco2, while natural extracts from juice residues (peels and seeds) were reported to be more effective than juice concentrates to induce a cell-cycle arrest in the same cells. Interestingly, this effect paralleled an increase of ROS in the cells, which suggests a ROS-induced cell death probably due to the pro-oxidant effects of the extracts. This pro-oxidant effect has been also reported for ovarian cancer cells by Feugang et al. [117], when compared to normal or immortalized cells. The use of pertinent controls, that is, cells of the same type with the same genetic background, is essential to conclude about the potentially beneficial effect of compounds. To be qualified as protective towards cancer, (phyto)-compounds need to be more cytotoxic to cancer cells than to their normal counterparts. In addition, Keller et al. [75] reported a protective effect of various *Opuntia* cladode flour towards the cytotoxic effect of 4-hydroxynonenal, a dietary lipid oxidation product possibly involved in the promoting effect of red meat on colon cancer. This protective effect was observed only on normal epithelial mouse colon cells, but not on the same cells bearing the Apc mutation, which is a frequent and early event in human colorectal carcinogenesis. Both normal and preneoplastic cells were immortalized cells obtained by crossing of normal or Min mice that carry the Apc mutation, with Immortomouse® mice.

However, in vivo studies are important to confirm those effects evidenced in vitro. Zou et al. [118] showed that *Opuntia* pear aqueous extracts suppressed tumor growth in nude mice, in an extent similar to the one these authors observed with the synthetic retinoid N-(4-hydroxyphernyl) retinamide (4-HPR) used as a chemopreventive model compound. A protective effect of *O. humifusa* was also reported by Hahm et al. on HeLa cells xenografts [115]. Some authors reported an effect of *O. ficus-indica* cladode extracts on oxidative stress and genotoxicity induced in vivo by the mycotoxins zearalenone and aflatoxin B1 [119–121]. Most of the time, *Opuntia* extracts were given intraperitoneally. More studies are needed to confirm the protective effects of *Opuntia* spp., testing those compounds in a more physiological way or for instance by oral route that will take into account the digestibility and bioavailability of such compounds. In this spirit, *O. humifusa* fruit lyophilized powder given in the pelleted diet was reported to be protective in two different animal models of skin carcinogenesis, together with a reduction of skin lipid peroxidation and skin inflammation [122, 123].

Taken together, all these studies show that *Opuntia* spp., as fruits, fruit juice, or nopal (*Opuntia* cladodes or stems), could provide an interesting anticancer strategy.

### 4.5. *Opuntia* spp. in Skin Wound Healing

As the largest organ of the human body and its location at the interface of the organism and the external environment, the skin has major protective properties, including a permeability barrier function, the maintenance of body temperature, and a role as a defense system against physical aggressions, ultraviolet (UV) radiations, microorganisms, and xenobiotics. The skin has also antioxidant and repair functions allowing removal of the damaged biomolecules, thereby preventing their accumulation and promoting wound healing.

The wound healing process is complex and fragile. It can be altered in various pathological situations (diabetes and arterial and metabolic diseases and infections and aging) and by multiple local and systemic factors among them (hypoxia and oxidative stress, decreased immune responses, infectious agents, inflammatory cytokines, metalloprotease activation, etc.); this leads to nonhealing chronic wounds [124]. Nopal and other *Opuntia* spp. extracts have long been used in traditional medicine for the treatment of burns, skin disorders, and wound healing, and the recent demonstration of their efficacy at the molecular and cellular levels justifies their use in nowadays dermatologic preparations [125].

Several recent studies point out the wound healing properties of *O. ficus-indica* cladode extracts. Using keratinocytes stimulated by benzopyrene or TNF-*α*, Nakahara et al. showed that *O. ficus-indica* cladode extracts may protect the epidermal barrier and the keratinocyte function by upregulating the expression of filaggrin and loricrin, two proteins present in differentiated keratinocytes and corneocytes. The protective effect of the extract is characterized by an inhibition of ROS production evoked by the inflammatory agents. This could result from the activation of the aryl hydrocarbon receptor, which in turn activates the transcription factor Nrf2 and subsequently the antioxidant NAD(P)H: quinone oxidoreductase 1 [126]. The cicatrizing properties of *O. ficus-indica* cladodes may involve both high molecular weight polysaccharide components such as a linear galactan polymer and a highly branched xyloarabinan, as well as low molecular weight components such as lactic acid, D-mannitol, piscidic, eucomic, and 2-hydroxy-4-(4′-hydroxyphenyl)-butanoic acids. These extracts could fasten cell regeneration on a scratched keratinocytes monolayer, suggesting that *O. ficus-indica* components exhibit high anti-inflammatory and high wound healing properties [127]. Likewise, polysaccharides extracted from cactus pear of *O. ficus-indica* stimulate the proliferation of fibroblasts and keratinocytes [128]. Among the protective agents present in the extracts, isorhamnetin glycoside components, such as diglycoside isorhamnetin-glucosyl-rhamnoside (IGR), could inhibit COX-2 and the production of TNF-*α* and IL-6 as well as the generation of nitric oxide (NO) evoked by lipolysaccharide (LPS) [129]. Moreover, cladode extracts from *O. humifusa* (Raf.) may regulate the production of hyaluronic acid (HA) by increasing the expression of HA synthase in keratinocytes exposed to UV-B treatment. Conversely, treatment using cladode extracts from *O. humifusa* could decrease the UV-B increased expression of hyaluronidase. Interestingly, the same protective effect on HA was observed in SKH-1 hairless mice exposed to UV-B, indicating that cladode extracts from *O. humifusa* have strong skin care capacities [130]. Altogether, these reports point out the ability of *Opuntia* spp. to accelerate wound healing and their potential interest as cotreatment of skin complications in diabetes and other pathologies characterized by a defective wound healing.

## 5. Conclusion and Future Directions

The vegetative parts of wild and domesticated *Opuntia* spp. have been used for centuries for nutritional and medicine purposes and are traditionally considered healthy nutritional sources for preventing chronic diseases such as diabetes, cardiovascular diseases, metabolic syndrome, obesity, or aging, as well as infectious or neurodegenerative diseases. Since several years, scientific research has been interested in deepening the knowledge on these plants, in order to better understand their nutritional and therapeutic properties. Moreover, there is also an interest in their development for economical purposes, as they easily grow in arid desert area. As reviewed in this article, a number of scientific studies have analyzed the *Opuntia* properties, particularly their phytochemical composition in fibers, antioxidants, vitamins, and protective peptides. Some of the biological processes evoked by the different *Opuntia* parts have been identified through in vitro studies and in vivo approaches, based on animal models and clinical trials. These studies emphasize their lipid-lowering, antidiabetic, and antiatherogenic properties, as well as their ability to slow down tumoral cell proliferation. Additional studies could be required to standardize the properties and the safety of *Opuntia* spp., knowing that their properties may differ as function of the different wild or domesticated species and the vegetative parts may exhibit variations in their phytochemical composition and properties. Nonetheless, it is likely that *Opuntia* spp. can be considered efficient functional food or nutraceuticals, able to prevent or slow down chronic disease development and promote a better health, quality of life, and longevity.

## Figures and Tables

**Table 1:** Chemical composition of wild and domesticated species in Mexico and other countries.

Species	Composition (%)				Phenolic acids^c^	Flavonoids^d^
	Protein	Fat	CF	Ash		
*O. streptacantha ^a^*	11.2	0.73	7.3	12.6	56.8	18.0
*O. hyptiacantha ^a^*	11.0	0.80	6.5	15.1	33.4	17.1
*O. megacantha ^a^*	10.7	0.69	6.5	13.6	44.7	16.8
*O. albicarpa ^a^*	11.6	0.75	6.5	13.2	40.8	17.2
*O. ficus-indica ^a^*	11.2	0.69	5.9	14.4	40.1	19.4
*O. humifusa^b^*	4.7	1.25	50.3	2.0	—	—

^a^Astello-Garcia et al. [28]; ^b^Jun et al. [131]; ^c^as mmol of gallic acid/g sample; ^d^as mmol of quercetin/g sample; CF: crude fiber.

**Table 2:** Mineral composition of wild and domesticated species in Mexico and other countries.

Species	Mineral (mg/100 g sample)					
	K	Ca	Na	P	Fe	Mn
*O. streptacantha ^a^*	2213	667	70	0.09	2.9	16.5
*O. hyptiacantha ^a^*	2690	740	87	0.09	3.9	9.8
*O. megacantha ^a^*	1960	683	137	0.08	5.1	13.3
*O. albicarpa ^a^*	1956	647	77	0.09	0.7	24.1
*O. ficus-indica ^a^*	2403	627	63	0.09	8.6	13.8
*O. humifusa^b^*	1269	1968	nd	1110	nd	1411

^a^Astello-Garcia et al. [28]; ^b^Jun et al. [131]; nd: not determined.

**Table 3:** Content of antioxidants compounds present in *Opuntia* species.

Species	Region	Tissue	Chemical compound	Concentration	Reference
*O. albicapa*	México	Pads (Cristalino cultivar)	Total phenolic acids	5.83–18 mg GAE/g DW	[22, 28]
			Total flavonoids	2.5–5.62 mg QE/g DW	[22, 28]
		Fruits (Reyna cultivar)	Total betalains	1 mg/100 g FW	[34]
			Ascorbic acid	1.8 mg/100 FW	[34]

*O. atropes*	México	Pads (Blanco cultivar)	Total phenolic acids	5.2 mg GAE/g DW	[22]
			Total flavonoids	9.7 mg QE/g DW	[22]

*O. dinellii*	Spain	Pads	Total phenolic acids	16.1 mg GAE/100 g FW	[52]
		Fruits	Total phenolic acids	117 mg QE/100 g FW	[35]
			Ascorbic acid	29.7 mg/100 g FW	[35]
	Taiwan	Fruits	Total phenolic acids	91 (juice) and 133 (peel) mg GAE/100 g FW	[132]
			Total flavonoids acids	32.5 (juice), 29.2 (peel) mg GAE/100 g FW	[132]
			Catechin	22.7 (juice), 18 (peel) mg/100 g FW	[132]
			Epicatechin	10.9 (juice), 17.1 (peel) mg/100 g FW	[132]
			p-Coumaric acid	0.6 (peel) mg/100 g FW	[132]
			Ferulic acid	4 mg/100 g FW (peel)	[132]
			Quercetin	4.6 mg/100 g FW (peel)	[132]
	Egypt	Fruit	Betacyanins	0.54 mg/100 mg DW	[36]
			Isorhamnetin-3-O-rutinoside	56 *μ*g/100 mg DW	[36]

*O. ficus*-*indica*	México	Pads	Total phenolic acids	6.8–18 mg GAE/g DW	[22, 28]
			Total flavonoids	5.3–6.1 mg QE/g DW	[28]
			Quercetin 3-O-rhamnosyl-(1-2)-[rhamnosyl-(1-6)]-glucoside	nq	[28]
	Spain	Pads	Total phenolic acids	128.8 mg GAE/100 g FW	[133]
	Italy	Fruits	Total phenolic acids	89.2 mg GAE/100 g FW	[40]
			Total betacyanins	39.3 mg/100 g FW	[40]
	Portugal	Fruit	Ferulic acid glucoside, piscidic acid, isorhamnetinpentosyl-rutinoside, isorhamnetinpentosyl-glucoside, isorhamnetinpentosyl-rhamnoside	nq	[42]

*O. hyptiacantha*	México	Pads	Total phenolic acids	5.39–6.14 mg GAE/g DW	[28]
			Total flavonoids	4.86–5.62 mg QE/g DW	[28]

*O. leucotricha*	Mexico	Pads (Duraznillo cultivar)	Total phenolic acids	3 mg GAE/g DW	[22]
			Total flavonoids	1.8 mg QE/g DW	[22]

*O. lindheimeri*	Mexico	Pads	Total phenolic acids	0.75 mg GAE/g DW	[134]
			Kaempferol	1.8 *μ*g/g DW	[134]
	USA	Fruits	Kaempferol	1.1 *μ*g/g FW	[37]
			Quercetin	90.5 *μ*g/g FW	[37]
			Isorhamnetin	1.9 *μ*g/g FW	[37]
			Ascorbic acid	121 *μ*g/g FW	[37]

*O. megacantha*	México	Pads	Total phenolic acids	6.7–19.5 mg GAE/g DW	[22, 28]
			Total flavonoid	3.2–5.62 mg QE/g DW	[22, 28]
		Fruit (Naranjona cultivar)	Total betalains	2.2 mg/100 g FW	[34]
	Argentina	Fruits	Total phenolic acids	36 mg GAE/100 g FW	[135]
			Total betalains	27 *μ*g/g FW	[135]
	Morocco	Fruits	Total flavonoids	50.24 *μ*g QE/g FW	[136]
			Total betalains	29.9 *μ*g/g FW	[136]

*O. rastrera*	Mexico	Pads	Total phenolic acids	0.39 mg/g DW	[134]
			Kaempferol	28.9 *μ*g/g DW	[134]
			Isorhamnetin	199.8 *μ*g/g DW	[134]
			Isorhamnetin-glucosyl-rhamnoside	nq	[134]
			Isorhamnetin + hexose + pentose	nq	[134]

*O. robusta*	México	Pads (Tapon cultivar)	Total phenolic acids	2 mg GAE/g DW	[22]
			Total flavonoids	3.8 mg QE/g DW	[22]
		Pads (Gavia cultivar)	Total phenolic acids	0.561 mg GAE/g DW	[134]
		Fruit (Camuesa cultivar)	Total betalains	6.8 mg/100 g FW	[34]
			Ascorbic acid	6 mg/100 g FW	[34]
		Pads (Tapon cultivar)	Total phenolic acids	0.39 mg GAE/g DW	[134]
			Kaempferol	45.6 *μ*g/g DW	[134]
			Isorhamnetin	99.58 *μ*g/g DW	[30]

*O. streptacantha*	Mexico	Pads	Total phenolic acids	0.66–11.07 mg GAE/g DW	[28, 134]
			Total flavonoids	4.92–5.74 mg QE/g DW	[28]
			Kaempferol	42.2 *μ*g/g DW	[134]
			Isorhamnetin	58.9 *μ*g/g DW	[134]
			Kaempferol 3-O-arabinofuranoside	nq	[28]
		Fruit (Cardona cultivar)	Total betalains	3.5 mg/100 g FW	[34]
	USA	Fruit (red-skinned)	Kaempferol	3.8 *μ*g/g FW	[37]
			Quercetin	51 *μ*g/g FW	[37]
			Ascorbic acid	815 *μ*g/g FW	[37]

*O. stricta*	Spain	Fruits	Total phenolic acids	204.4 GAE/100 g FW	[33]
			Total betalains	80.1 mg/100 g FW	[33]
			Ascorbic acid	23.3 mg/100 g FW	[33]
			Quercetin	87.5 *μ*g/g FW	[33]
			Isorhamnetin	50.3 *μ*g/g FW	[33]
			Kaempferol	7.7 *μ*g/g FW	[33]
			Luteolin	15.6 *μ*g/g FW	[33]
	USA	Fruits	Total flavonoids	9.8 *μ*g/g FW	[37]
			Quercetin	9.8 *μ*g/g FW	[37]
			Ascorbic acid	437 *μ*g/g FW	[37]

*O. undulata*	Mexico	Pads	Total phenolic acids	0.95 mg GAE/g DW	[134]
			Kaempferol	12.9 *μ*g/g DW	[134]
			Isorhamnetin	326.9 *μ*g/g DW	[134]
			Isorhamnetin-glucosyl-rhamnosyl-rhamnoside	nq	[134]
			Isorhamnetin + 1 hexose + 1 methylpentose + pentose	nq	[134]
			Kaempferol-glucosylrhamnoside	nq	[134]
	Spain	Fruits	Total phenolic acids	164.6 mg GAE/g FW	[33]
			Total betalains	42.4 mg/100 g FW	[33]
			Total flavonoids	51.1 *μ*g/g FW	[33]
			Quercetin	30 *μ*g/g FW	[33]
			Isorhamnetin	9.6 *μ*g/g FW	[33]
			Kaempferol	5.6 g/g FW	[33]
			Luteolin	5.9 *μ*g/g FW	[33]

*O. violacea*	México	Pads (Morado cultivar)	Total phenolic acids	20 mg GAE/g DW	[22]
			Total flavonoids	3.5 mg QE/g DW	[22]

nq: not quantified.

## Abbreviations

4-HPR: N-(4-Hydroxyphernyl) retinamide
AGIs: *α*-Glucosidase inhibitors
BMI: Body mass index
CAD: Cardioartery diseases
CAM: Crassulacean acid metabolism
FW: Fresh weight
GAE: Gallic acid equivalents
GIP: Glucose-dependent insulinotropic peptide
GLP-1: Glucagon-like peptide 1
HDL: High-density lipoprotein
HNE: 4-Hydroxy-2-nonenal
ICAM-1: Intercellular adhesion molecule
LDL: Low-density lipoprotein
LOX-1: Lectin-like oxidized low-density lipoprotein receptor-1
LPO: Lipid oxidation products
MDA: Malondialdehyde
NO: Nitric oxide
NOX2: NADPH oxidase 2
*O.*: *Opuntia*
OGTT: Oral glucose tolerance test
*Opuntia* spp.: *Opuntia* species
QE: Quercetin equivalent
ROS: Reactive oxygen species
STZ: Streptozotocin
T2DM: Type 2 diabetes mellitus
VCAM-1: Vascular cell adhesion protein 1
VLDL: Very low-density lipoprotein.
